# Supplementary material for: From command-line bioinformatics to bioGUI
Source: PeerJ. 2019 Nov 21;7:e8111. doi: 10.7717/peerj.8111 (PMC6875409; doi:10.7717/peerj.8111)
Supplement: Supplemental Information 1 — The original survey used to assess problems with current bioinformatics applications. [file peerj-07-8111-s001.pdf]

Bioinformatics Tools Usage

Hello,

welcome to the Bioinformatics Tools Usage study.

This study consists of 4 sections:

1) General bioinformatics tools questions
2) Specific bioinformatics tools questions
3) Hands-On study 1
4) Hands-On study 2

Please answer the questions in 1+2 first, then proceed to question 3 and 4.

Your answers will be recorded anonymously.

Thank you for your participation,

Markus

* Required

Command-Line Interface vs Graphical User Interface

When talking about command-line tools we refer to programs/tools which are run only from the command-line. This particularly means you have to enter each command as text and you cannot use your mouse to select files or navigate along the file system.

When talking about programs with a graphical user interface we think about programs, which allow most tasks to be performed with a mouse. This might be selecting files to open (see screenshot), but might also refer to more complex tasks.

Command-line Interface (example)

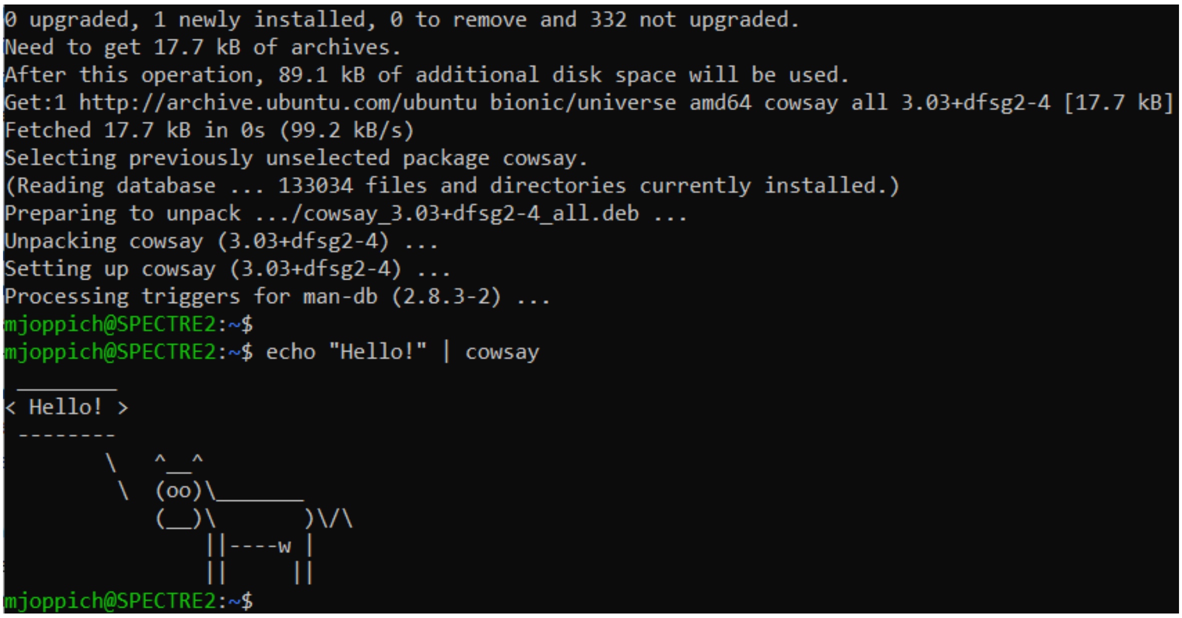

Graphical User Interface (example)

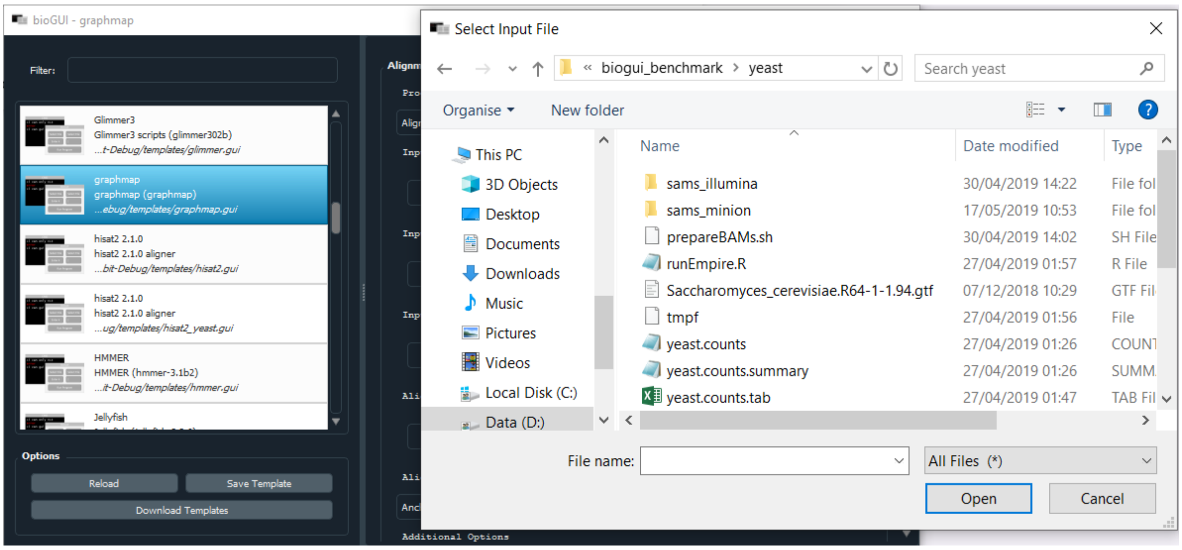

1. **In which field of study do you have most experience?** *

Mark only one oval.

- ( ) Medicine
- ( ) Biology
- ( ) Chemistry
- ( ) Bioinformatics
- ( ) Other: ____________________

2. **What are common tasks where you would like to have assistance from bioinformatics tools or where you use them already? Where could these assist your (better)?** *

3. **Have you recently tried to use or used bioinformatics tools?** *

Mark only one oval.

- ( ) Yes
- ( ) No

4. **If you have used bioinformatics tools, which ones and for which task?** *

5. **Are there other available tools for your task?** *
Mark only one oval.

- ⬭ Yes
- ⬭ No
- ⬭ I don't know

6. **For which of the following reasons did you choose this/these tools?** *
Check all that apply.

- [ ] command-line interface
- [ ] graphical user interface
- [ ] only tool available for this task
- [ ] it's the industry standard
- [ ] only tool provided by employer
- [ ] easy to use
- [ ] recommended by colleague
- [ ] recommended by specialists (e.g. bioinformaticians)
- [ ] Other: ______________________

7. **How would you rate your computer skills regarding the usage of software?** *
How confident are you in using software? If you are presented a new software, how fast do you get an idea of how to use this software?
Mark only one oval.

	1	2	3	4	5	
Novice	⬭	⬭	⬭	⬭	⬭	Expert

8. **Do you have any programming skills (e.g. R, python, ...)?** *
You should select "No programming skills" when you never had to write an R script, or python script, etc. for your job. Please do not consider Excel as a programming language.
Mark only one oval.

	1	2	3	4	5	
No programming skills	⬭	⬭	⬭	⬭	⬭	I am an expert programmer

9. **Have you ever used a command-line program?**
Mark only one oval.

- ⬭ Yes
- ⬭ No

10. **How easy would it be for you to use the command-line?** *

Here we are interested in your priorities. How much effort would it be for you to use the command-line in order to run a tool? If you do not feel distracted at all, choose "Not much/1". If you think you could manage to run a program from the command-line, but feel you could do more relevant tasks to your job, choose "3". If you think you could not use command-line tools, choose "Unable to cope with/5".

Mark only one oval.

	1	2	3	4	5	
Not much	⬭	⬭	⬭	⬭	⬭	Unable to cope with

11. **If you think about using bioinformatic tools, would you generally prefer** *

Check all that applies. If you prefer a GUI for some cases, and a CLI for other cases, check both.

Check all that apply.

- [ ] a graphical user-interface (GUI)
- [ ] a command-line interface (CLI)
- [ ] depends on the task/use-case
- [ ] Other: ____________________

12. **Why did you choose the answer above?** *

____

Your experience with bioinformatics tools (1)

Please answer these questions with regard to a bioinformatics tool you used or wanted to use (does not mean you successfully used it!).

With bioinformatics tool we refer to both open-source tools, (antiSMASH, glimmer, hisat2, ...) and commercial systems like Genious or CLC workbench.

13. **Please decide for a bioinformatic tool you recently used or wanted to use? If this is not applicable to you, skip this section!** *

____

14. **Is this tool a commercial tool or freely available?** *

Mark only one oval.

- ( ) Commercial/Proprietary
- ( ) Free/Open Source
- ( ) Other: _______________

15. **Why did you choose to use this tool?**

Check all that apply.

- [ ] easy-to-use
- [ ] has a graphical user interface (GUI)
- [ ] has a command-line interface (CLI)
- [ ] solves my problem
- [ ] only available software to me to solve a specific task
- [ ] no alternative known
- [ ] recommended by colleague
- [ ] recommended by specialist
- [ ] Other: _______________

16. **Have you been able to run/use the tool (no matter whether it answered your scientific question)?** *

Mark only one oval.

- ( ) Yes
- ( ) No

17. **Would you like to use the tool again?** *

Mark only one oval.

- ( ) Yes
- ( ) No

18. **Which kind of user-interface does the tool have?** *

Mark only one oval.

- ( ) Graphical-User Interface (Window/GUI)
- ( ) Command-line Interface (Console/Terminal only)
- ( ) Other: _______________

19. **If you have not been able to run the tool, why could you not run the tool? Please check all answers that apply.** *

Check all that apply.

- [ ] I could not download the software
- [ ] The software requires another operating system
- [ ] After downloading the software, I did not know how to install it
- [ ] After installing the software, I did not know how to use the software
- [ ] I could not use the software because there was no graphical-user interface
- [ ] I could not use the software because there was no command-line interface
- [ ] Other: ______________________

In the following section we will ask you how easy you found specific processes during the use of the bioinformatic tool.

If you could not access a step (e.g. you could not run the tool, because you could not install it), please choose the "Does not apply" option.

20. **Downloading/Accessing the software was easy.** *

Mark only one oval.

- ( ) Strongly disagree
- ( ) Disagree
- ( ) Neutral
- ( ) Agree
- ( ) Strongly agree
- ( ) Does not apply

21. **Installing the software was easy for me.** *

Mark only one oval.

- ( ) Strongly disagree
- ( ) Disagree
- ( ) Neutral
- ( ) Agree
- ( ) Strongly agree
- ( ) Does not apply

22. **Using the included graphical-user interface was easy?** *

Mark only one oval.

- ( ) Strongly disagree
- ( ) Disagree
- ( ) Neutral
- ( ) Agree
- ( ) Strongly agree
- ( ) Does not apply/No GUI

23. **Using the included command-line interface was easy?** *

Mark only one oval.

- Strongly disagree
- Disagree
- Neutral
- Agree
- Strongly agree
- Does not apply/No CLI

24. **What were the most struggle-some tasks in accessing and using the software?** *

Check all that apply.

- downloading the software
- installing the software
- checking that all dependencies are installed (in the right location)
- starting the software
- using the software
- finding the settings/options needed
- Other: ______________________________

Hands-on Use-Case 1

In this use-case we want to find out how easy it is for you to use a tool on the command line.

We want you to install graphmap and align long reads to a given reference.

In preparation for this study, make sure to have downloaded the provided archive to the test computer and extract it into a folder "study1" in your home directory.

Step 1: Download and install graphmap

Abort this step after 5 minutes, no matter how far you have gotten.

We want to download and install graphmap (https://github.com/isovic/graphmap).

A Terminal has been opened for you. You are now in the 'study1' folder. Enter the following commands to install graphmap.

git clone https://github.com/isovic/graphmap.git
cd graphmap

and enter

make modules
make -j 4

cd ..

Following this, run

./graphmap/bin/Linux-x64/graphmap

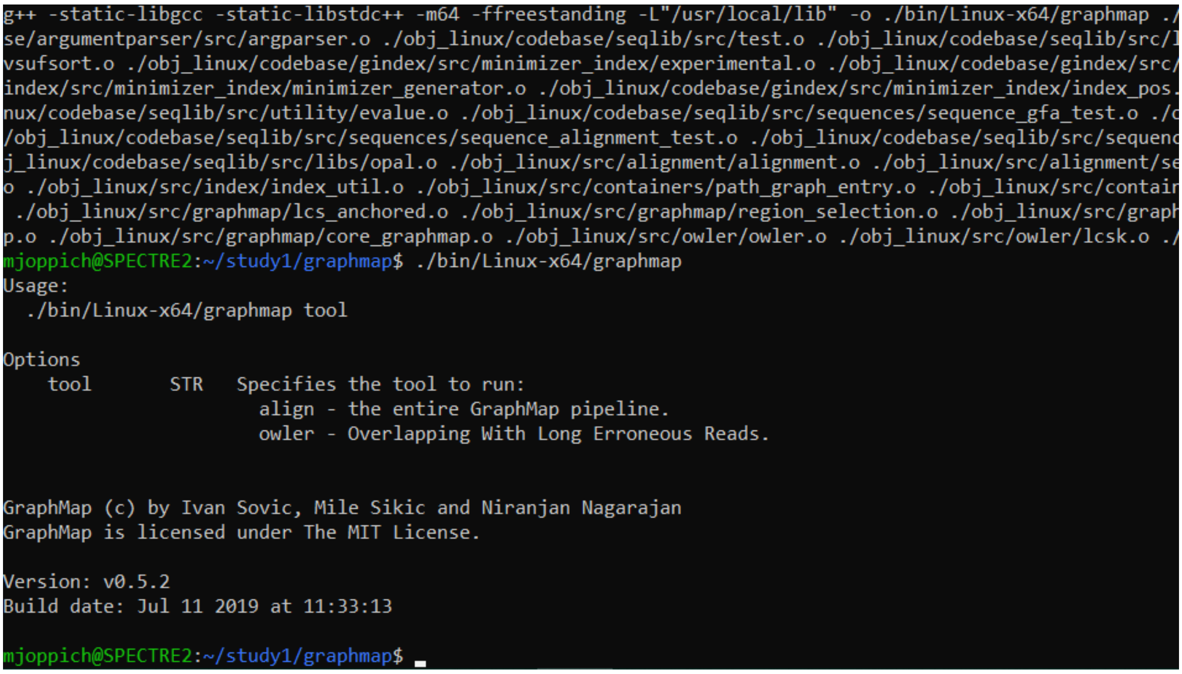

25. **How long did you take?** *

Example: 8:30 AM

26. **Did you successfully install graphmap?** *

You have been successful if the output of the last command looks similar to the screenshot above.

Mark only one oval.

- Yes
- No

27. **Has the installation process been easy?** *

Mark only one oval.

	1	2	3	4	5	
No						Yes

28. **How easy would it have been for you to install graphmap WITHOUT the given help?** *

Mark only one oval.

	1	2	3	4	5	
No						Yes

Step 2: Using graphmap on the command line

Abort this step after 5 minutes, no matter how far you have gotten.

We now have to align the reads to a reference.

Within the study1 folder there are 2 files. Enter 'ls<Enter>' in the Terminal to see them:
scer_genome.fa
SRR5989373.short.fastq

These are the input files for the alignment. scer_genome.fa is the reference genome and SRR5989373.short.fastq are the reads.

Try to find out how to align the reads. The output file should be

mapped.sam

in the study1 folder.

29. **How long did you take?** *

Example: 8:30 AM

30. **Did you successfully start the alignment?** *

Mark only one oval.

- Yes
- No

31. **What was your command?** *

32. **Has it been easy to align the reads?**

e.g. commands were clearly explained, etc.

Mark only one oval.

	1	2	3	4	5	
No	⬭	⬭	⬭	⬭	⬭	Yes

33. **What were your main challenges installing and using graphmap?**

____

____

____

____

____

Hands-on Use-Case 2

In this use-case we want to find out how easy it is for you to use a tool with bioGUI.

We want you to install graphmap and align long reads to a given reference.

In preparation for this study, make sure to have downloaded the provided archive to the test computer and extract it into a folder "study1" in your home directory.

Abort this task after 5 minutes, no matter how far you have gotten.

Step 1: Install graphmap

Abort this step after 5 minutes, no matter how far you have gotten.

We want to download and install graphmap (https://github.com/isovic/graphmap).

Locate the study1 folder and the bioGUIRelease subfolder.

Double-click on the bioGUI icon (in the bioGUIRelease subfolder) and start bioGUI.

Click on "Download Templates" and download the "Install graphmap (github)" template.

Select the "Install Template Module".

Before installing graphmap, check the following settings:
- If you are running bioGUI on Windows check "Use WSL?"
- You want to install graphmap into your user data directory
- You do not have a User password/sudo rights
- Whether or not graphmap is available in your PATH does not matter
- You want to save the generated template with name "graphmap"

Install the program using bioGUI.

Once completed, check whether bioGUI has installed graphmap and "Reload".

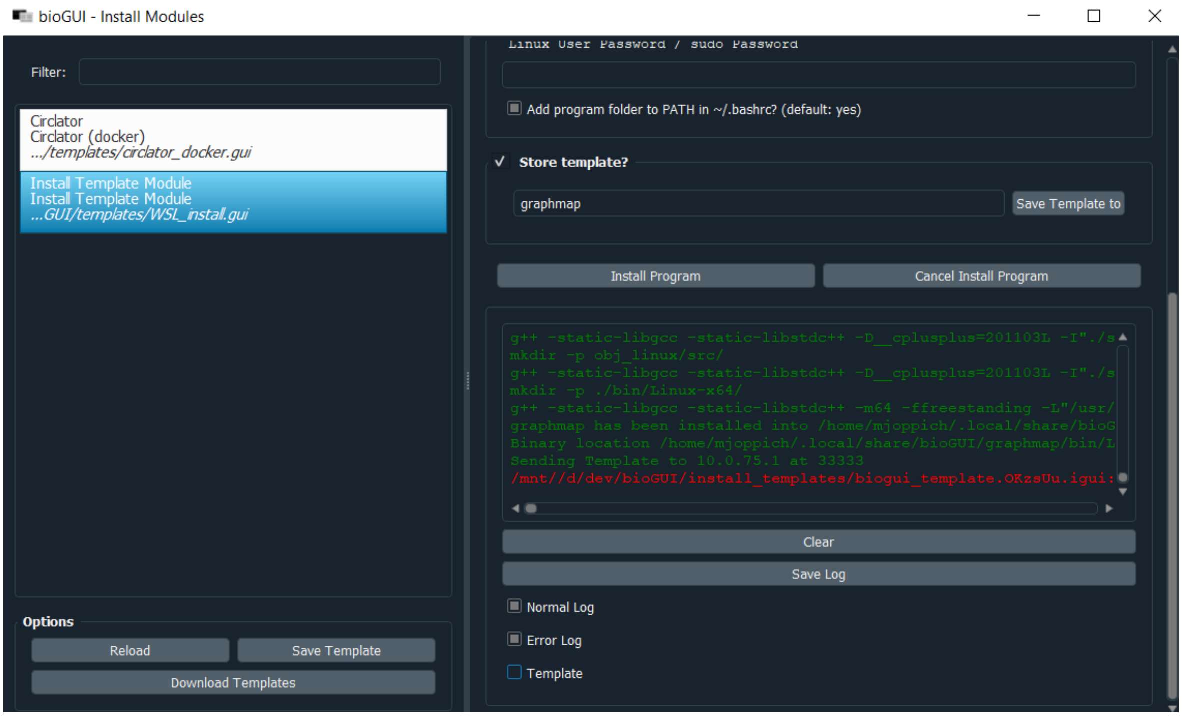

34. **How long did you take?** *

Example: 8:30 AM

35. **Did you successfully install graphmap?** *

Mark only one oval.

- ( ) Yes
- ( ) No

36. **Has the installation process been easy?** *

Mark only one oval.

	1	2	3	4	5	
No	( )	( )	( )	( )	( )	Yes

37. **Would you have been able to install graphmap without the given help?** *

Mark only one oval.

	1	2	3	4	5	
No	( )	( )	( )	( )	( )	Yes

Step 2: Use graphmap

Abort this step after 5 minutes, no matter how far you have gotten.

We now have to align the reads to a reference.

Within the study1 folder there are 2 files:
scer_genome.fa
SRR5989373.short.fastq

These are the input files for the alignment. scer_genome.fa is the reference genome and SRR5989373.short.fastq are the reads.

Try to find out how to align the reads. The output file should be

mapped.sam

in the study1 folder.

38. **How long did you take?** *

____

Example: 8:30 AM

39. **Did you successfully start the alignment?** *

Mark only one oval.

- ( ) Yes
- ( ) No

40. **Has it been easy to align the reads?**

e.g. options were clearly explained, etc.

Mark only one oval.

	1	2	3	4	5	
No	( )	( )	( )	( )	( )	Yes

41. **What were your main challenges installing and using graphmap?**

You are almost done!

After this experience, which interface would you prefer for the given task ...

42. **... for a single/few alignment runs?**

Mark only one oval.

	1	2	3	4	5	
Command-Line	◯	◯	◯	◯	◯	GUI

43. **... for quite some alignment runs?**

Mark only one oval.

	1	2	3	4	5	
Command-Line	◯	◯	◯	◯	◯	GUI

44. **Overall: Which interface was easier to use in your opinion?**

Mark only one oval.

	1	2	3	4	5	
Command-Line	◯	◯	◯	◯	◯	GUI

45. **In this test: what were disadvantages of bioGUI for you?**

____

____

____

____

____

46. **In this test: what were advantages of bioGUI for you?**

____

____

____

____

____

Powered by
Google Forms
